# Supplementary material for: Digital Health Innovations for Screening and Mitigating Mental Health Impacts of Adverse Childhood Experiences: Narrative Review
Source: JMIR Pediatr Parent. 2024 Oct 16;7:e58403. doi: 10.2196/58403 (PMC11498064; doi:10.2196/58403)
Supplement: Multimedia Appendix 1 [file pediatrics-v7-e58403-s001.docx]

| References | Purpose | Study type | ACE Risk Factor | Author’s conclusion |
| --- | --- | --- | --- | --- |
| Liverpool et al (2020) [23] | To (1) identify modes of delivery used in children and young people digital MH interventions; (2) explore factors influencing usage and implementation; and (3) investigate ways in which the interventions have been evaluated and whether children and young people engages in DHIs. | Systematic Review | Mental Health Illness | Findings suggest the development and use of digital health solutions are increasing significantly and may be of interest to children and young people, particularly around mental health treatment. |
| Lehtimaki et al (2021) [24] | To examine current evidence on digital health interventions targeting adolescents and young people with mental health conditions, with a focus on effectiveness, cost-effectiveness, and generalizability to low-resource settings. | Systematic Review | Mental Health Illness | Conclusion suggests only a small proportion of existing digital platforms are evidence based and demonstrate low effectiveness in low- and middle-income countries. As such, widespread adoption and scale-up of digital mental health interventions will require more rigor in research and study. |
| Punukollu and Marques (2019) [25] | To evaluate currently available literature concerning the use of online mobile-based applications and interventions in the detection/management/maintenance of young people’s mental health and well-being. | Systematic Review | Mental Health Illness | Findings suggest mental health apps directed at young people have the potential to be important assessment/management/treatment tools, therefore creating easier access to health services, helping in the prevention of mental health issues, and capacitating to self-help in case of need. |
| Aschbrenner et al (2019) [26] | To examine how adolescents receiving public mental health services use digital technology and social media; to explore their preferences using technology to support health and wellness. | Parallel Convergent Mixed Methods | Mental Health Illness | Findings suggest that digital health interventions to promote health and wellness among adolescents may be scalable and strongly effective in community mental health settings. |
| Valentine et al (2019) [27] | To present a summary of current research regarding the impact of social networking sites (SNS) on the health and well-being of young people. | Systematic Review | Mental Health Illness | Findings argue social networking sites (SNS) may be leveraged to revolutionize children and young people’s access to/engagement with therapeutic digital interventions, improving mental health outcomes for children and young people overall. |
| Fullagar et al (2017) [28] | To offer a new approach to analyzing interrelations between formal/informal pedagogical sites for learning about mental health in youth with a specific focus on digital health technologies. | Systematic Review | Mental Health Illness | Conclusions suggest the use of individual mental health apps developed with a broader digital ecology offers crucial tools to mental health studies, public pedagogy and digital sociology converge around the problem of youth mental health and illness. |
| Lekkas and Jacobson (2021) [30] | To examine the ability to utilize Global Positioning System (GPS) data, derived passively from a smartphone across seven days, to detect PTSD diagnostic status among a cohort (N = 185) of high-risk, previously traumatized women. | Retrospective Cohort | PTSD | Findings suggest utility of GPS information as a digital biomarker of the PTSD behavioral repertoire may prove vital in detection outside traditional clinical contexts. Future PTSD research will benefit from applications of GPS data within larger, more diverse populations. |
| Chen and Chan (2022) [32] | To provide an overview and understanding of the effectiveness of digital health interventions to prevent and reduce the occurrence of unintentional injury, violence, and suicide. | Meta-analysis/Systematic Review | Suicide | Conclusions suggest digital health interventions have great potential to reduce unintentional injury, violence, and suicide. Future research should explore DHIs' successful components to facilitate future implementation and wider access. |
| Carson et al (2019) [33] | To study the rapid proliferation of machine learning research using electronic health records to classify healthcare outcomes offers an opportunity to address the pressing public health problem of adolescent suicidal behavior | Retrospective Cohort | Suicide | Conclusion suggests that the use of machine learning approach and natural learning process has moderate success in identifying suicide attempts in a small sample of hospitalized pediatric psychiatric division. |
| Su et al (2020) [34] | To investigate that built-in machine learning models can predict suicidal behavior among children and adolescents by collecting the data from their longitudinal clinical records, and determining short- and long-term risk factors | Retrospective Cohort | Suicide | Conclusions suggest predictive models of suicide risk of children and adolescents by using demographics, comorbidity diagnosis codes, laboratory test results, and medications from clinical records models show good performances for estimation of short-term and long-term risks and identified significant predictors which may assist in clinical practices. |
| Walsh et al (2018) [35] | To study the validation of machine learning approach on longitudinal clinical data in adults to discuss the challenges in adolescents | Retrospective Longitudinal Cohort | Suicide | Conclusion suggests machine learning on longitudinal clinical data may provide a scalable approach to broaden screening for risk of nonfatal suicide attempts in adolescents. |
| Khanna and Carper (2022) [37] | To review current literature on digital mental health interventions (web-/cloud-based programs, mobile applications, virtual reality, etc.) and digital assessment methods such as ecological momentary assessment for the delivery and/or support of evidence-based care in child anxiety. | Systematic Review | Anxiety | Findings suggest that current research is promising for the use of digital mental health interventions to improve access and efficiency of evidence-based practice. However, ethics and practice guidelines are needed, and questions remain regarding what level and quality of therapeutic involvement is needed to maximize treatment/ensure positive outcomes in youth. |
| Williams and Pykett (2022) [38] | To outline the extent of the clinical evidence base of mental health apps with monitoring functions for depression/anxiety in children and young people; categorize the range of monitoring features/understand their various purposes; and analyze these ‘technical mechanisms’ in apps from the perspective of critical ecological analysis. | Scoping Review | Anxiety | Conclusions suggest that the examination of the bioethics and neuroethics of digital health technologies is necessary and urgent. This requires paying closer attention to the social practices of technology-enabled self-monitoring and the ways in which these frames mental health as a form of individualized emotional regulation. |
| Fried et al (2022) [39] | To investigate the usefulness of digital mindfulness-based interventions as a promising strategy for managing anxiety in children and young people. | Prospective Cohort | Anxiety, ADHD | Findings suggest children with comorbid anxiety and/or sleep problems and/or ADHD could benefit from a digitally based meditation application. |
| Pandian et al (2021) [41] | To compare game based digital therapeutic devices to digital health interventions to treat ADHD along with pharmacology and behavioral therapy | Systematic Review | ADHD | Findings suggest that game-based digital therapeutic devices, such as EndeavorRx, are strongly recommended for the treatment of inattentive or combined-type ADHD who have demonstrated an attention issue. |
| Kollins et al (2020) [42] | To assess whether AKL-T01 (Software Treatment for Actively Reducing Severity of ADHD) improved attentional performance in pediatric patients with ADHD | Randomized, double-blind, parallel group, controlled clinical trial | ADHD | Conclusions suggest that AKL-T01 provides positive evidence for improving ADHD in pediatric age groups. |
| Ammar et al (2021) [45] | To present the Semantic Platform for Adverse Childhood  Experiences Surveillance (SPACES) - an explainable multimodal  AI platform to facilitate surveillance of adverse experiences and diagnosis of related health conditions and develop subsequent interventions. This study utilizes a bottom-up approach to multimodal, explainable knowledge graph-based learning to derive recommendations/insight for better resource allocation/care management. | Semantic networking - advanced knowledge representation, constructing knowledge graphs with multimodal features; populating graphs with data  from multi-dimensional datasets. | Mental Health Illness | SPACEs provide a novel approach to ACEs surveillance/screening/diagnosis and subsequent management of related medical conditions by providing a 360-degree data landscape that integrates community/socio-environmental contextual knowledge with  individual-level/local knowledge. |
| Brenas et al (2019) [47] | To implement a formal ontology as a resource to allow the mental health community to facilitate data integration and knowledge modeling; to improve ACEs’ surveillance/research. | Semantic networking - advanced knowledge representation utilizing semantic Web tools and techniques to implement ontologies | Mental Health Illness | Conclusion suggest ACEs Ontologies provide a reliable semantic network/integrated knowledge structure for mental health practitioners and researchers to employ in work to improve ACE surveillance and evaluation. |

**a.** ADHD, attention-deficit/hyperactivity disorder; AKL-T01, Software Treatment for Actively Reducing Severity of ADHD; GPS, global positioning system; PTSD post-traumatic stress disorder; DHI, digital health intervention; SNS, social networking sites; ACEs, adverse childhood experiences; SPACES, semantic platform for adverse childhood
